# Supplementary material for: On the influence of cell shape on dynamic reaction-diffusion polarization patterns
Source: PLoS One. 2021 Mar 18;16(3):e0248293. doi: 10.1371/journal.pone.0248293 (PMC7971540; doi:10.1371/journal.pone.0248293)
Supplement: S1 File — (DOCX) [file pone.0248293.s001.docx]

**On the influence of cell shape on dynamic diffusion-reaction polarization patterns**

Eroumé K.^1^, Vasilevich A.^2^, Vermeulen S^1,2^, de Boer J.^2^, Carlier A.^1^

^1^ MERLN Institute for Technology-inspired Regenerative Medicine, Maastricht University, Universiteitssingel 40, 6229 ER, Maastricht, the Netherlands

^2^ Dept. of Biomedical Engineering, Eindhoven University of Technology, Eindhoven, the Netherlands.

1. **Extended model**

**Model use and access**

We have provided a link to the standard polarization model which is available as a public model in the VCell repository as Kerbai_PLoSone_2021_teardrop_polarization

_extended for the extended model and Kerbai_PLoSone_2021_teardrop_polarization

_minimal for the minimal model under the user name KerbaicBITE. Model codes (extended and minimal) are provided in the supplementary (S28_Models).Details on how to run a model in VCell can be found in the quick start guide on the VCell website, <https://vcell.org/support>.

**Geometry definition**

An analytic expression was used to define the symmetric shapes (circle, rectangle and square) in Virtual Cell. This can be specified in the geometry definition menu of the current model. The asymmetric shapes (teardrop, wide drop, and triangle) were created by importing images of those shapes into Virtual Cell (see S29). The narrow drop was created by reducing the height (or minor axis) of the teardrop from 20 µm to 5 µm. All other asymmetric shapes had a height (or minor axis) of 20 µm. For every shape the various domains, cell, nucleus and extracellular regions were then defined. The major axis length was set at 50 µm and kept constant across the shapes. The Each domain was finally mapped to its corresponding region and named accordingly in the structure mapping menu of Virtual Cell. Further details of the geometry definition process can be found in the “quick start guide” or in one of the tutorials at <https://vcell.org/support>.


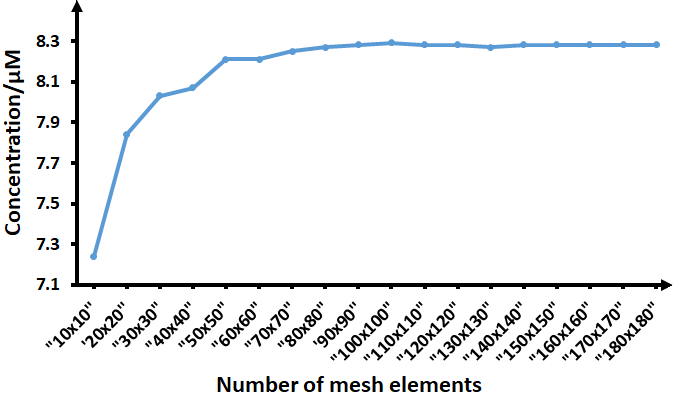


**S1 Fig.** **Mesh convergence analysis. Total Cdc42 concentration at t = 500s for different mesh elements.** Case of the teardrop at standard settings


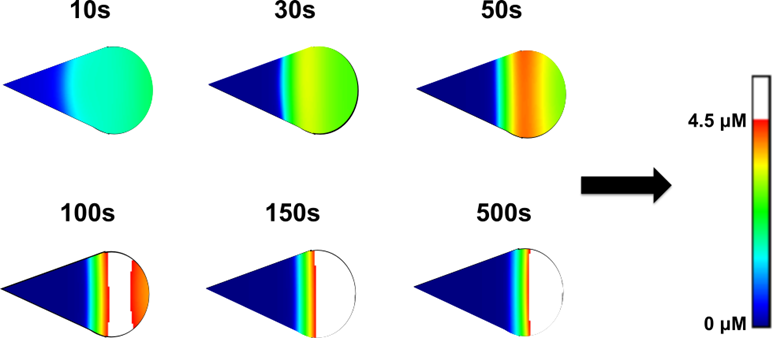


**S2 Fig.** **Spatiotemporal evolution of active Rac in a teardrop-shaped cell. Regions where the active Rac concentration is above 4.5 µM are colored white.** The black arrow indicates the initial left-right direction of polarization


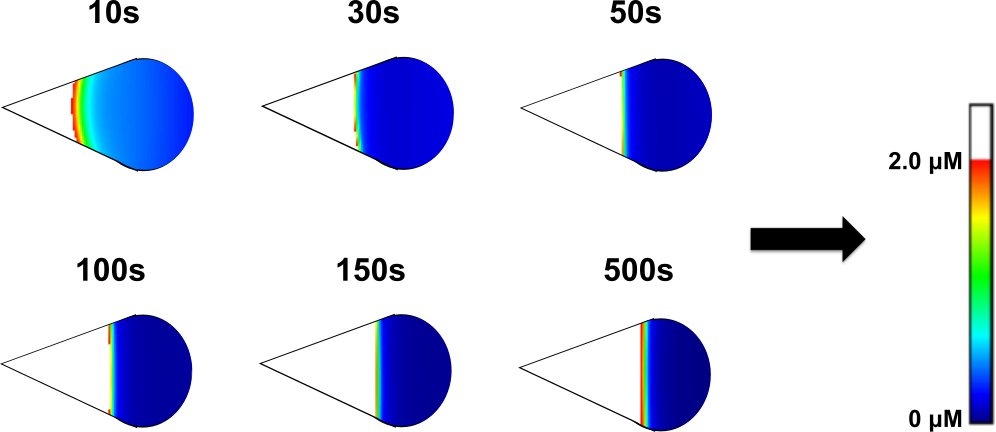


**S3 Fig.** **Spatiotemporal evolution of active Rho in a teardrop-shaped cell.** Regions where the active Rho concentration is above 2.0 µM are colored white. The black arrow indicates the initial left-right direction of polarization


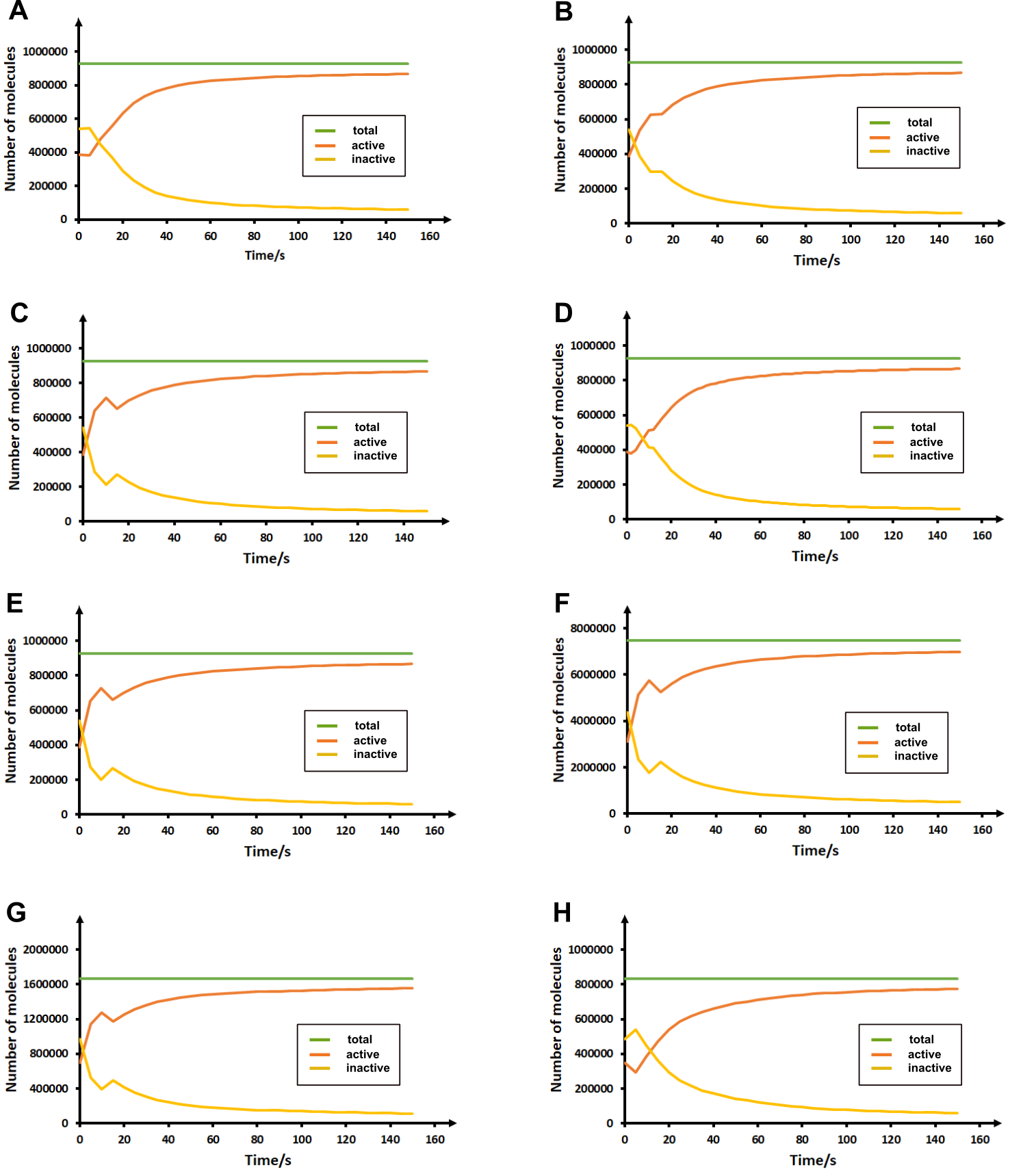


**S4 Fig.** **Conservation of Cdc42 molecules (y-axis) over time in the extended model.** Teardrop initial Cdc42 activation A) Ic = 1.6 + 0.05x, B) Ic = 2.6 + 0.05x, C) Ic = 3.6 + 0.05x, D) Ic = 2.6 + 0.025x, E) Ic = 2.6 + 0.1x and aspect ratios F) 90x90 µm, G) 90x20 µm, H) 10x90 µm


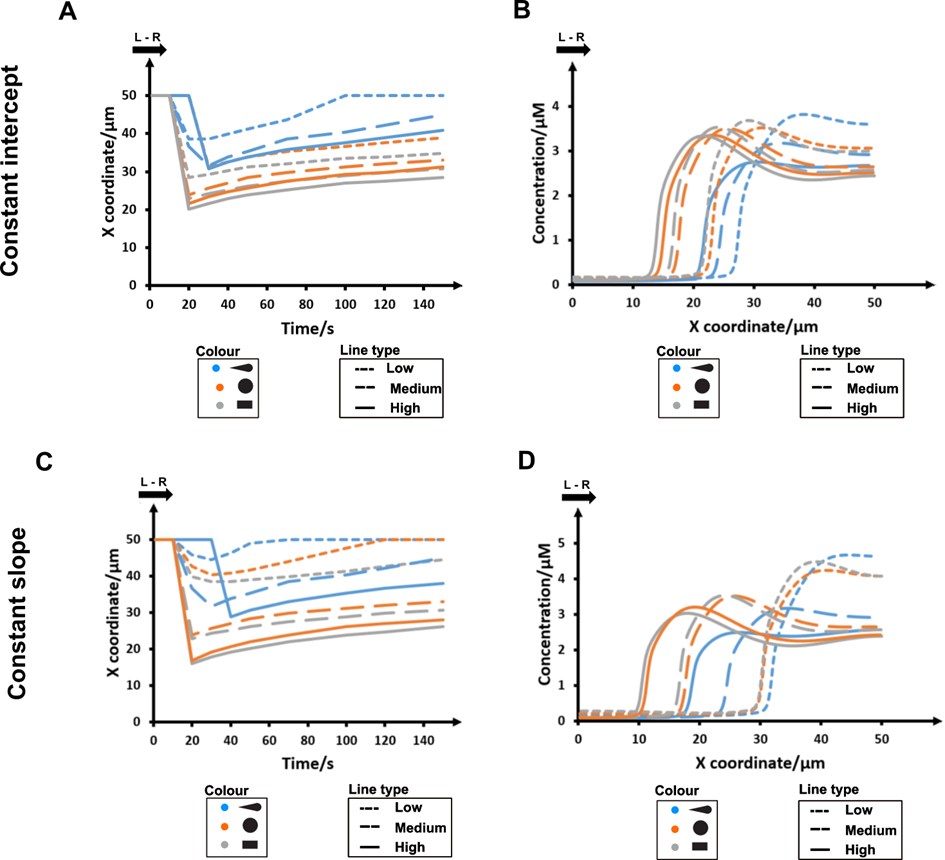


**S5 Fig.** **Initial polarization strength influences the reverse polarization pattern.** Constant intercept of 2.6, slope values: low=0.025, medium=0.05, high= 0.1. Constant slope of 0.05, intercept values: low=1.6, medium=2.6, high=3. The line color represents the cell shape while the line type represents the level of the initial polarization parameter. A) Temporal evolution of maximal active Cdc42 concentration at constant intercept. B) Spatial evolution of active Cdc42 concentration at 30 s for constant intercept. The concentrations are measured on the main axis. C) Temporal evolution of maximal active Cdc42 concentration at constant slope. D) Spatial evolution of active Cdc42 concentration at 30 s at constant slope. The concentrations are measured on the main axis. In D the “high” circle and rectangle overlap. The initial polarization direction is left-right as indicated by the black arrow.


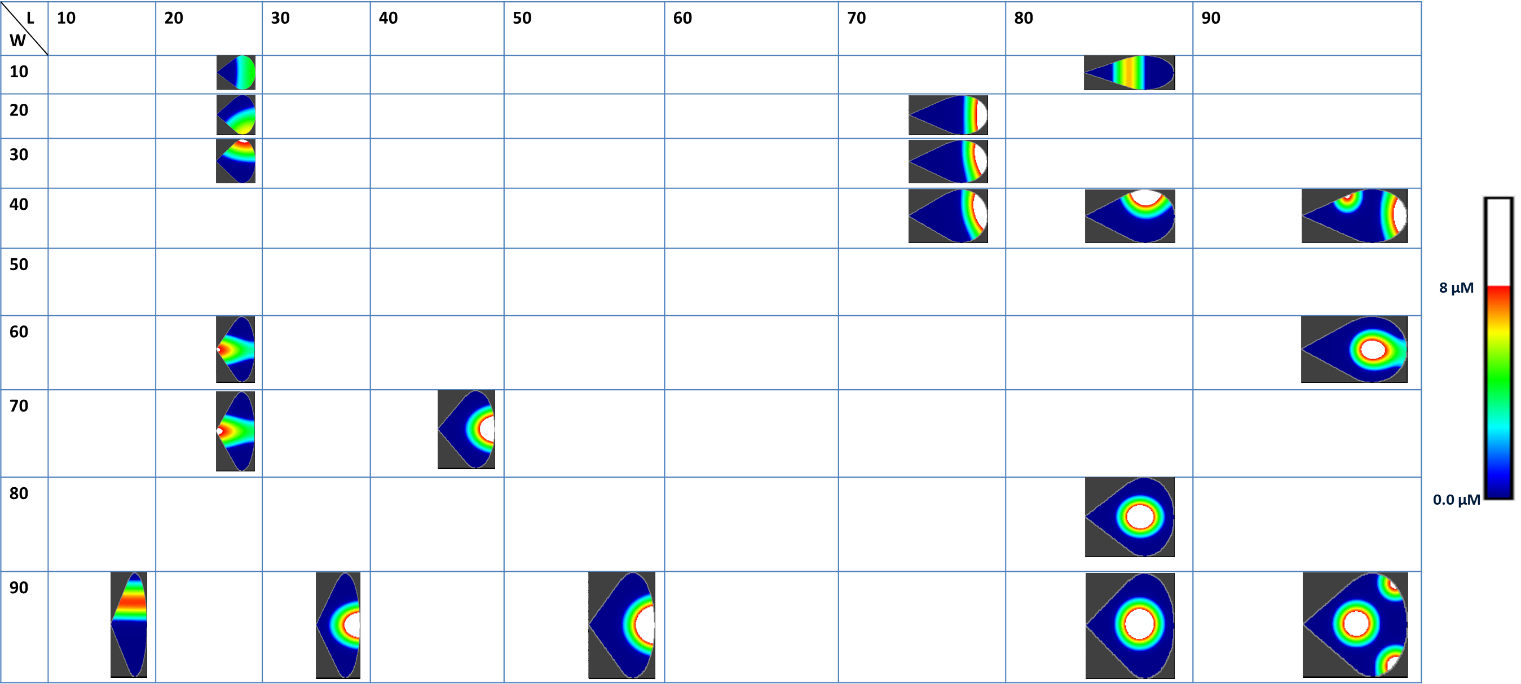


**S6 Fig.** **Cell size and aspect ratio affect polarization patterns in the teardrop, simulation time = 3000 s.**


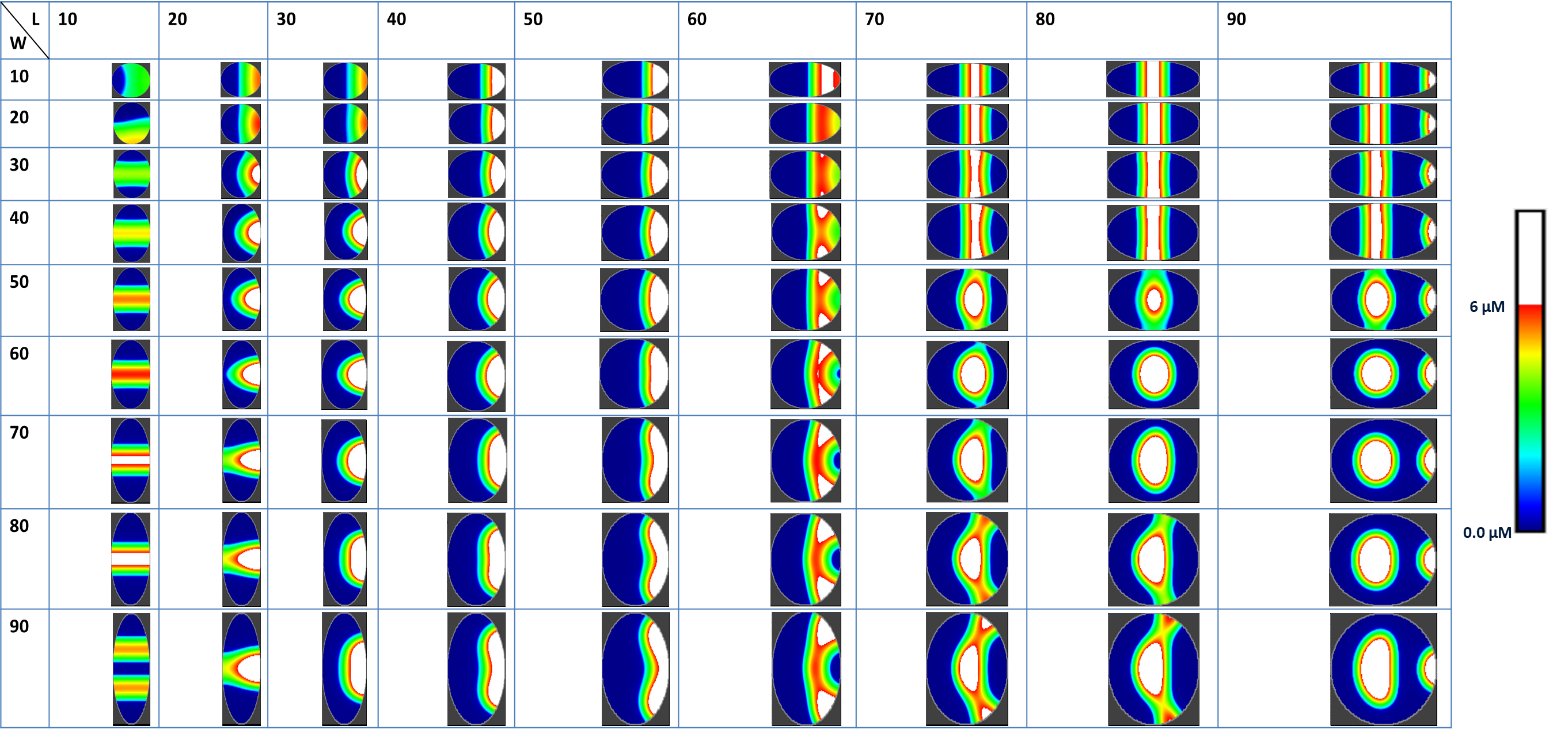


**S7 Fig.** **Cell size and aspect ratio affect polarization patterns in the circle, simulation time = 1000 s.**


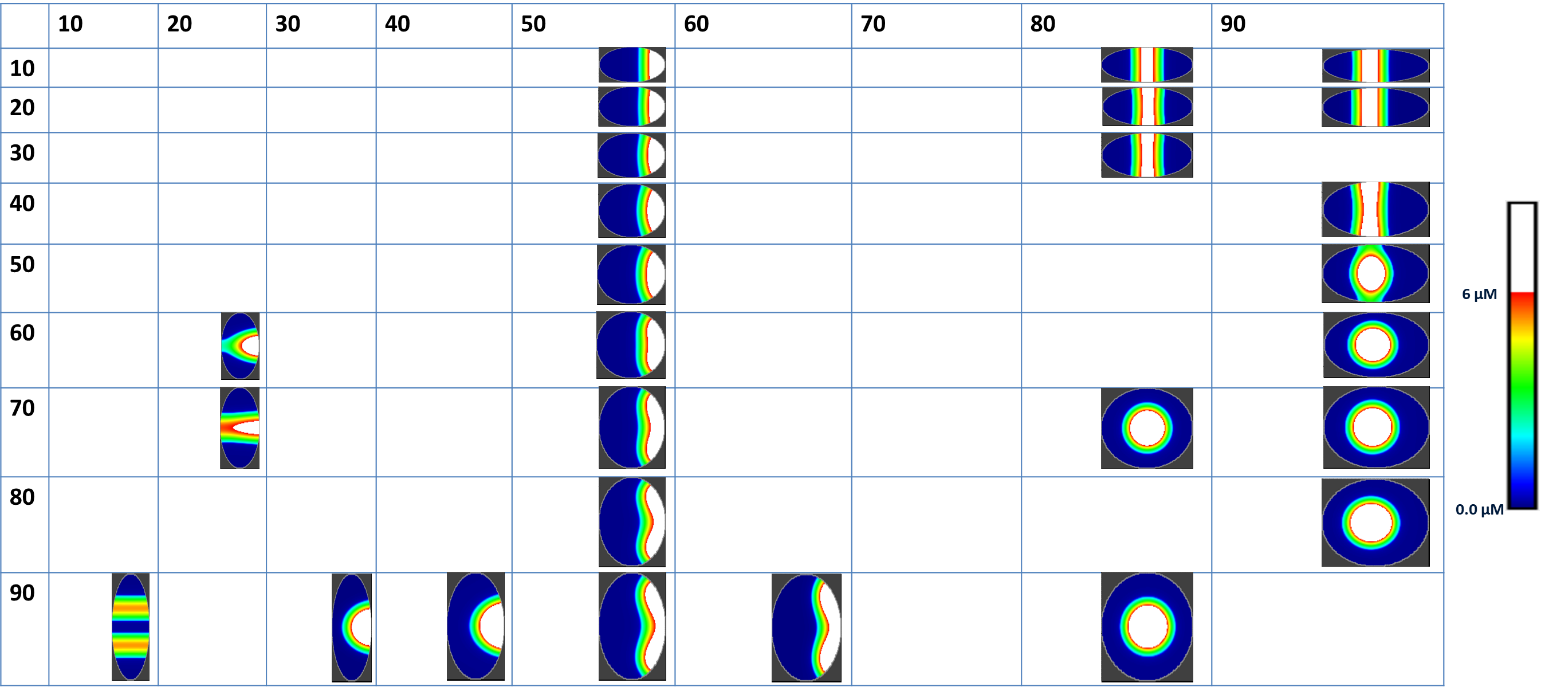


**S8 Fig.** **Cell size and aspect ratio affect polarization patterns in the circle, simulation time = 3000 s.**


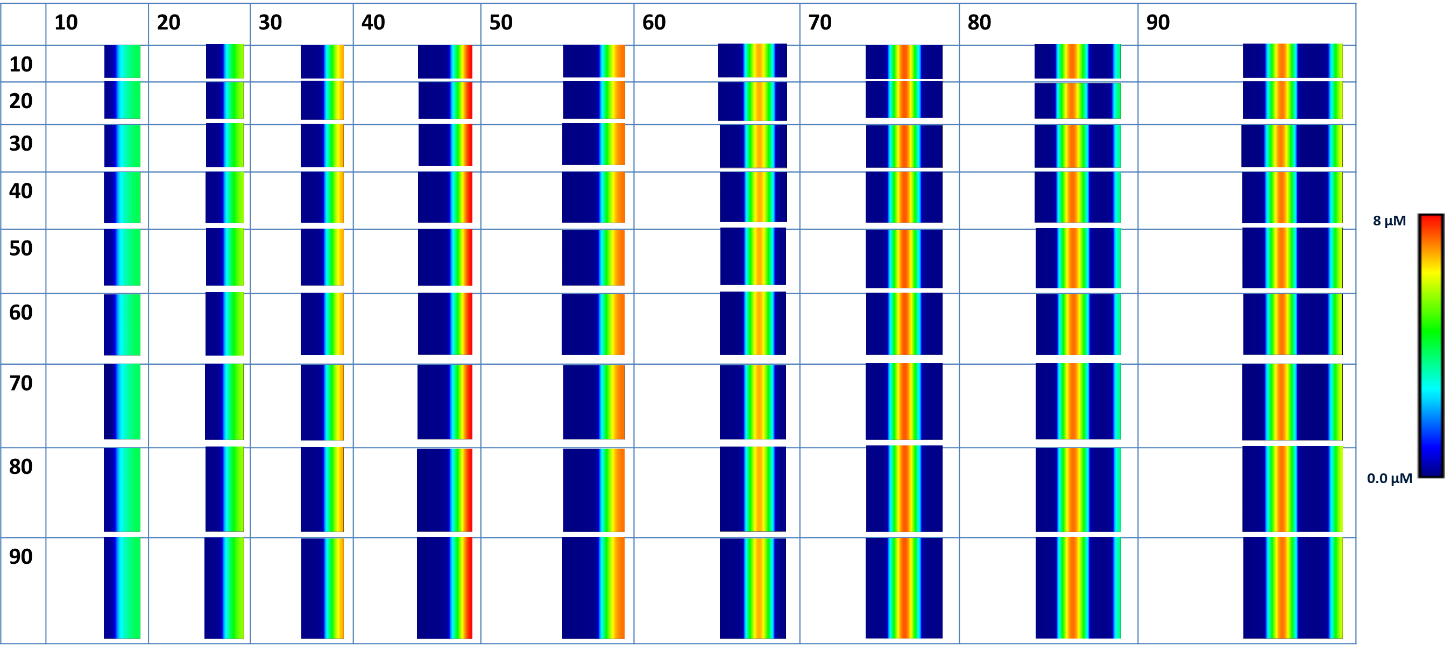


**S9 Fig.** **Cell size and aspect ratio affect polarization patterns in the rectangle, simulation time = 1000s.**


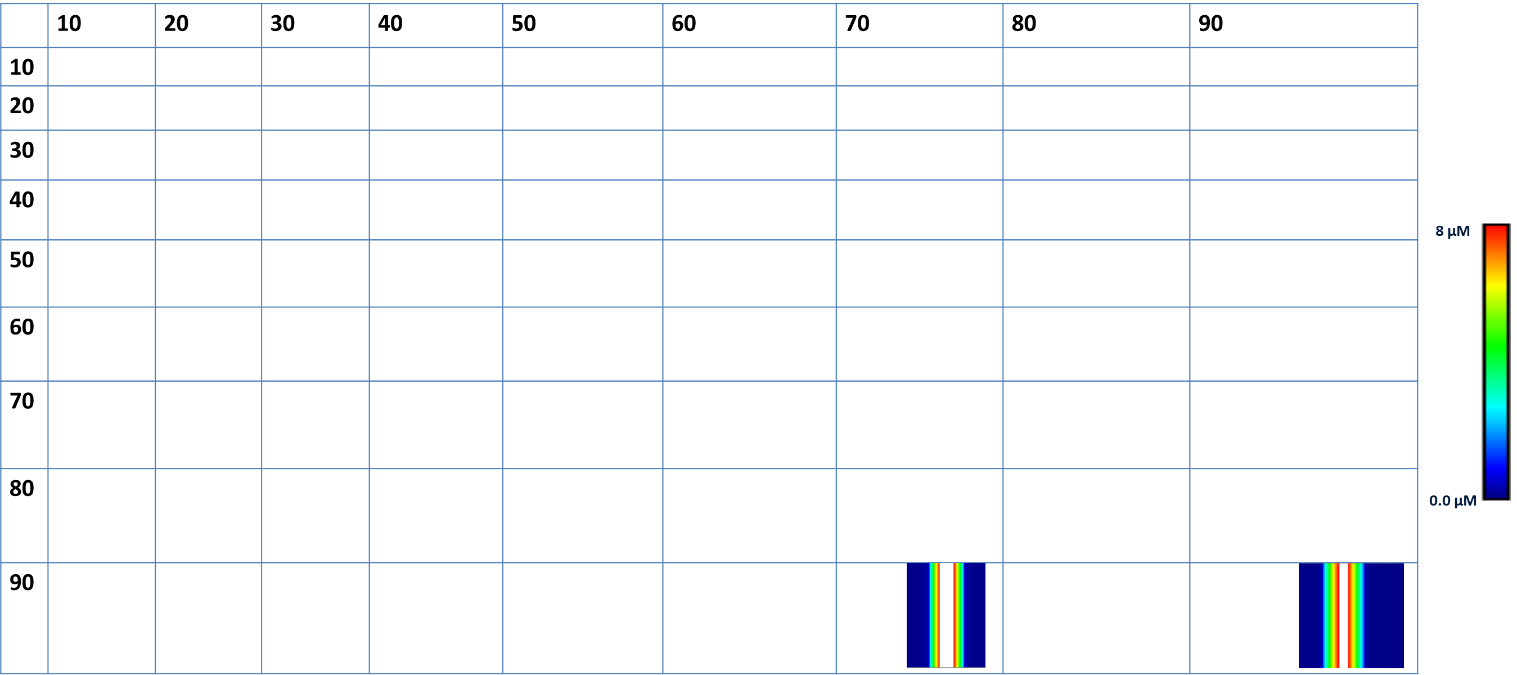


**S10 Fig.** **Cell size and aspect ratio affect polarization patterns in the rectangle, simulation time = 3000 s**


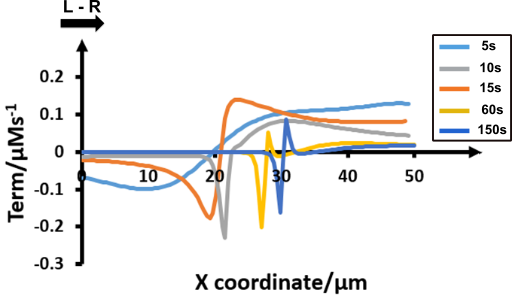


**S11 Fig.** **Spatial evolution of the “activation-inactivation” term at standard settings for the teardrop (with trends at 60 s and 150 s shown).** The concentrations are measured on the main axis. The black arrow indicates the initial left-right direction of polarization


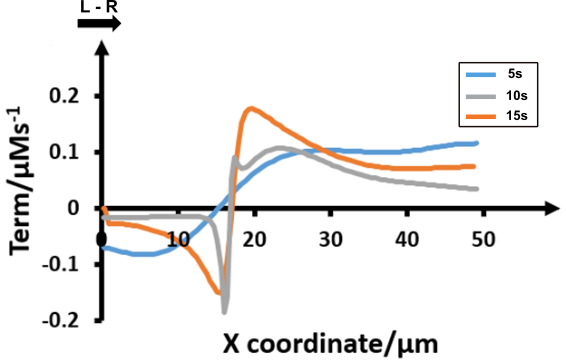


**S12 Fig.** **Spatial evolution of the “activation-inactivation” term at standard settings for the circle.** The concentrations are measured on the main axis. The black arrow indicates the initial left-right direction of polarization


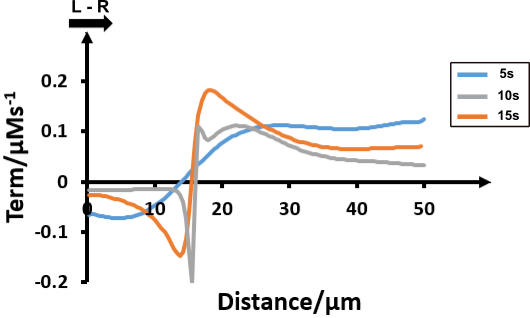


**S13 Fig.** **Spatial evolution of the “activation-inactivation” term at standard settings for the rectangle. The concentrations are measured on the main axis.** The black arrow indicates the initial left-right direction of polarization


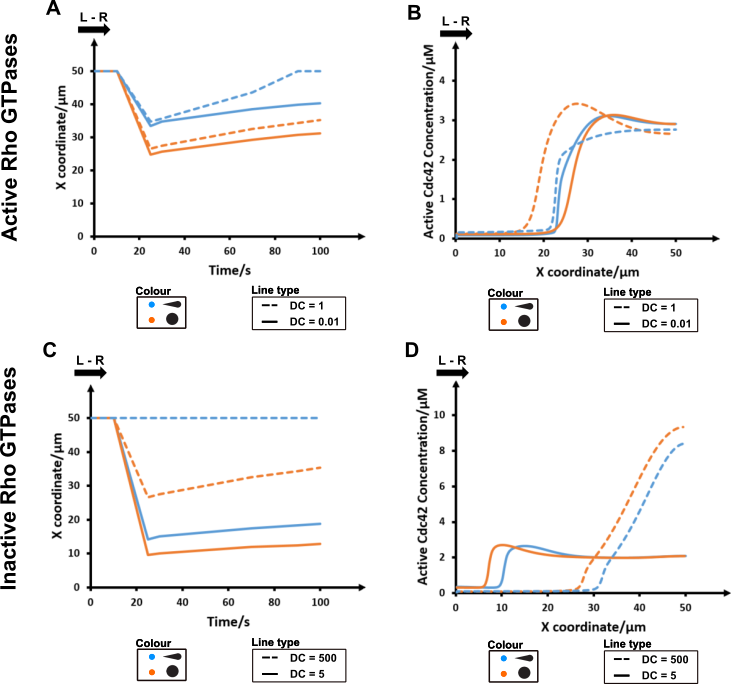


**S14 Fig.** **Influence of the diffusion coefficients (DC) of active (A and B) and inactive Rho GTPases (C and D).** A) Temporal evolution of the maximal active Cdc42 concentration and B) Spatial evolution of active Cdc42 concentration at t = 30 s for DC values (0.01, 1) of active Rho GTPases. C) Temporal evolution of the maximal active Cdc42 concentration and D) Spatial evolution of active Cdc42 concentration at t = 30 s for DC (5, 500) of inactive Rho GTPases.

**
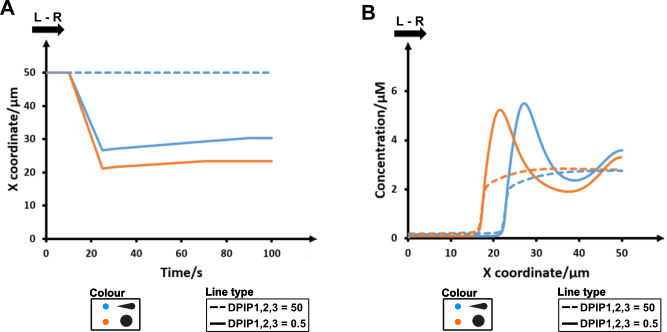
**

**S15 Fig.** **Influence of the diffusion coefficients (**DPIP_1_, DPIP_2_, DPIP_3_**) of Phosphoinisitides (PIPs).** A) Temporal evolution of the maximal active Cdc42 concentration and B) Spatial evolution of active Cdc42 concentration at t = 30s for DPIP_1_, DPIP_2_, DPIP_3_ values (0.5, 50).


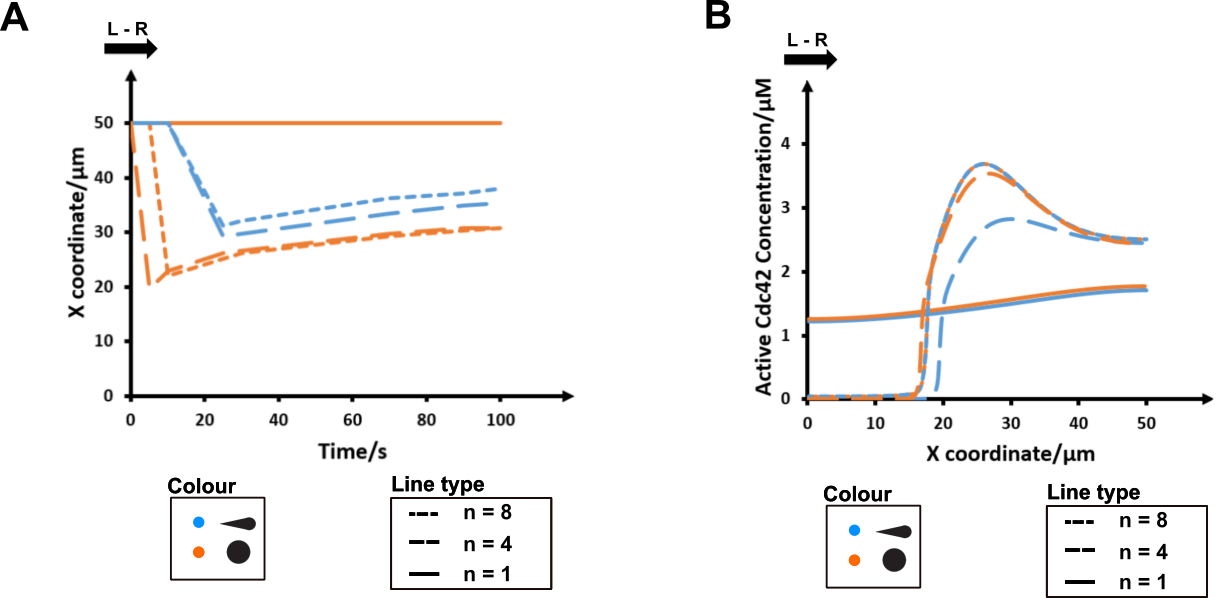


**S16 Fig.** **Influence of the Cdc42-Rho mutual inhibition parameter (n).** A) Temporal evolution of the maximal active Cdc42 concentration and B) Spatial evolution of active Cdc42 concentration at t = 30s.


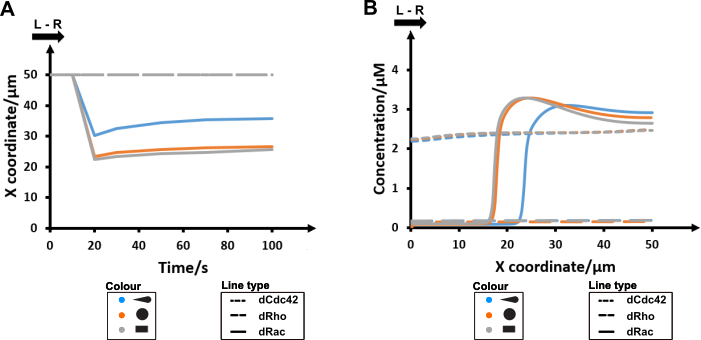


**S17 Fig.** **Influence of the inactivation rate parameter values (drac = 0.01, d_cdc42_ = 0.01 and d_rho_ = 0.01).** A) Temporal evolution of the maximal active Cdc42 concentration. The graphs of dCdc42 and dRho overlap for all the shapes. B) Spatial evolution of active Cdc42 concentration at t = 30 s. The concentrations are measured on the main axis. The graphs of dCdc42 overlap for the circle and the rectangle, while the graphs of dRho overlap for all shapes. The black arrow indicates the initial left-right direction of polarization


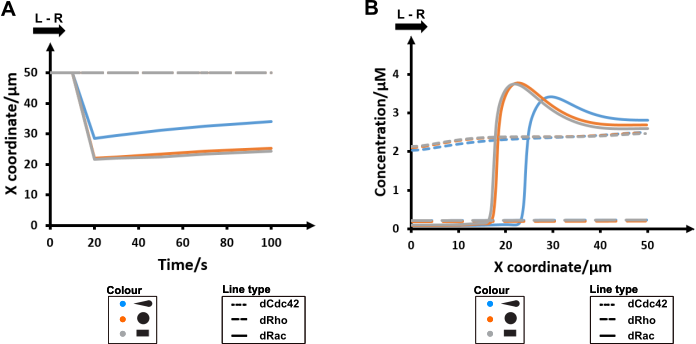


**S18 Fig. Influence of the inactivation rate parameter values (d_rac_ = 0.1, d_cdc42_ = 0.1 and d_rho_ = 0.1).** A) Temporal evolution of the maximal active Cdc42 concentration. The graphs of d_Cdc42_ and d_Rho_ overlap for all the shapes. B) Spatial evolution of active Cdc42 concentration at t = 30 s. The concentrations are measured on the main axis. The graphs of d_Cdc42_ overlap for the circle and the rectangle while the graphs of d_Rho_ overlap for all shapes. The initial polarization direction is left-right, as indicated by the black arrow.


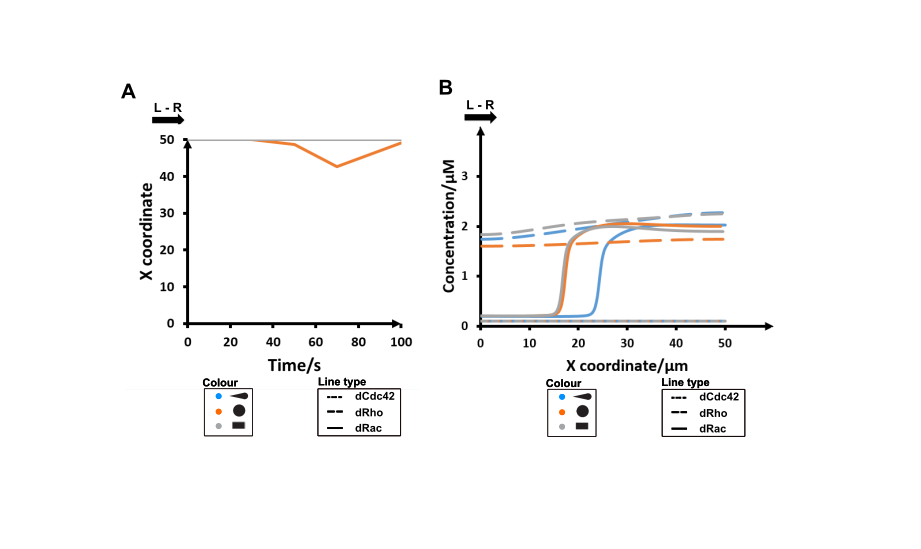


**S19 Fig.** **Influence of the inactivation rate parameter values (d_rac_ = 10, d_cdc42_ = 10 and d_rho_ = 10).** A) Temporal evolution of the maximal active Cdc42 concentration. The graphs of d_Cdc42_, d_Rho_ and d_Rac_ overlap for all the shapes, the exception being the graph of d_Rac_ for the circle which shows reverse polarization. B) Spatial evolution of active Cdc42 concentration at t = 30 s. The concentrations are measured on the main axis. The graphs of dCdc42 overlap for the circle and the rectangle while the graphs of dRho overlap for all shapes.The black arrow indicates the initial left-right direction of polarization


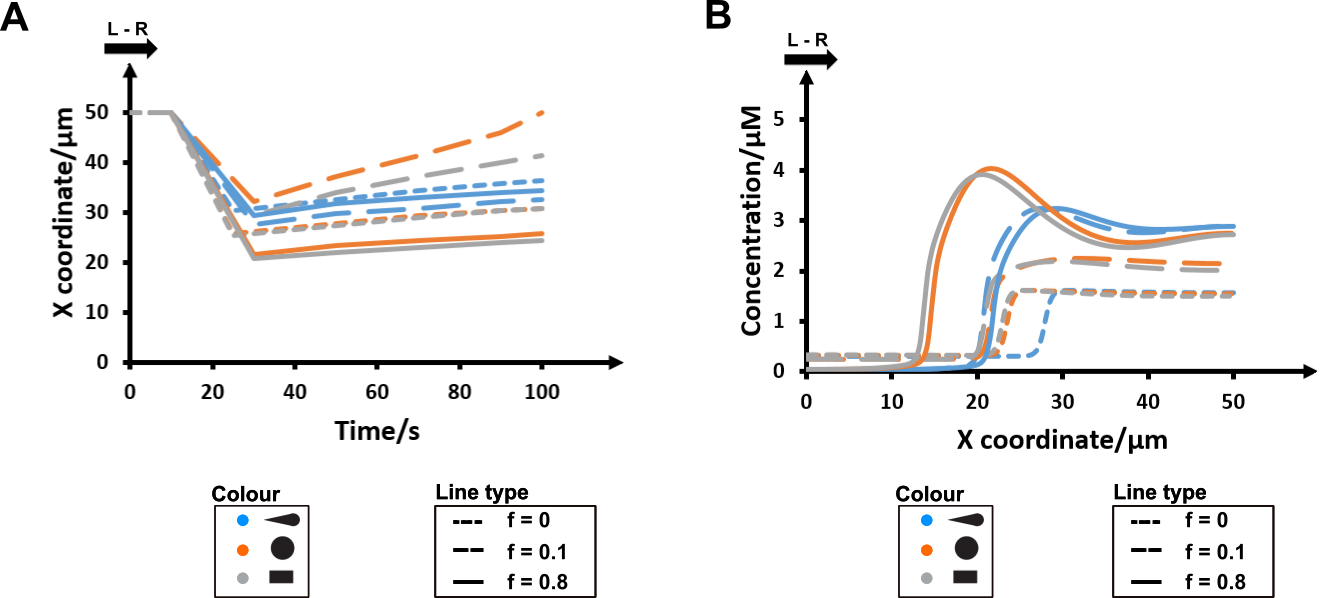


**S20 Fig.** **Influence of the f parameter on cell polarization.** A) Temporal evolution of the maximal active Cdc42 concentration. B) Spatial evolution of active Cdc42 concentration at 30 s. The concentrations are measured on the main axis. The initial polarization direction is left-right, as indicated by the black arrow.


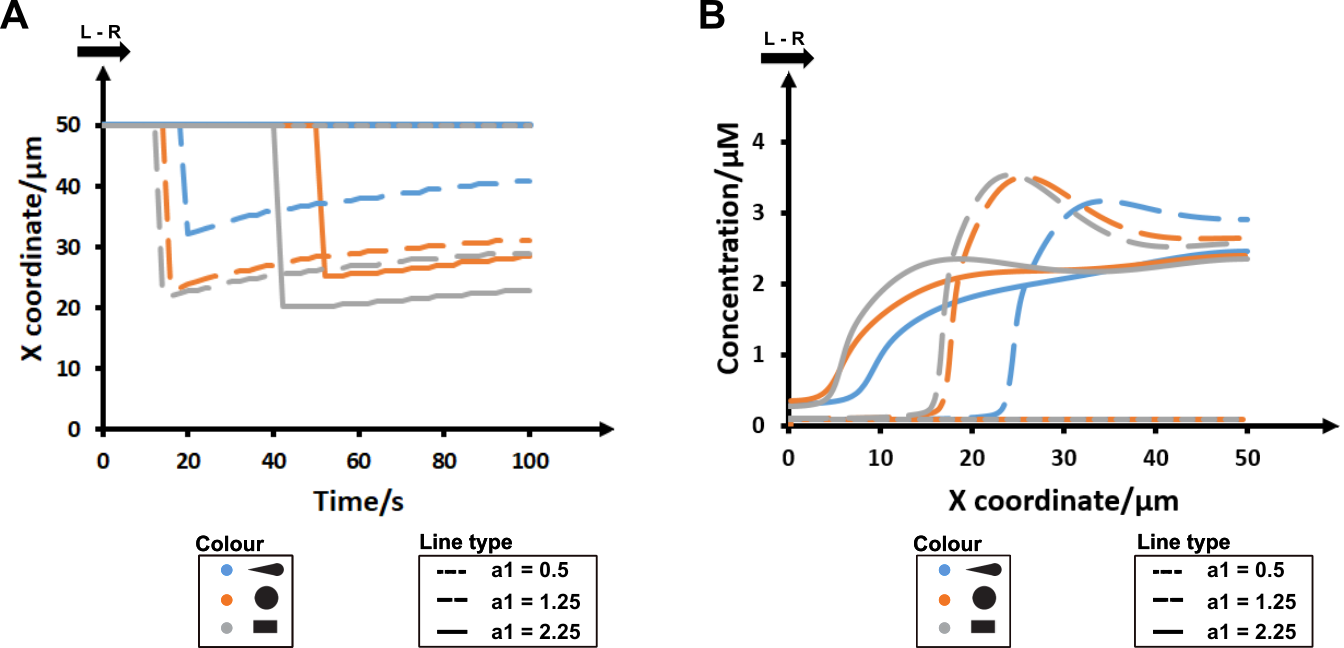


**S21 Fig.** **Influence of the a_1_ parameter on cell polarization.** A) Temporal evolution of the maximal active Cdc42 concentration. The graph of a_1_ = 2.25 of the teadrop is a straight line at the 50μm mark, which overlaps with parts of other curves at different time points. B) Spatial evolution of active Cdc42 concentration at 30s. The concentrations are measured on the main axis. The initial polarization direction is left-right, indicated by the black arrow.

1. **Minimal model**


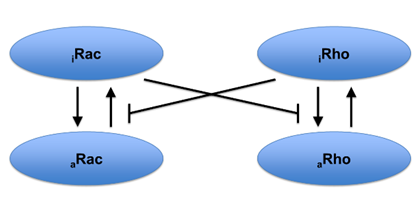


**S22 Fig. Schematic overview of the minimal Rho GTPase model adapted from** [1]**.** The subscripts i and a refer to the inactive and active Rho GTPases respectively.

- 1. ***Model equations***

The schematic representation of the minimal Rho GTPase model is shown in Fig. S15. Here we have four PDEs which we slightly modified from the model in [1]. Here $R$ and $\rho$ represent the levels of active Rac and Rho, $R_{i}$ and $\rho_{i}$ represent the levels of inactive Rac and Rho, and $R_{tot}$ and $\rho_{tot}$ represent the total Rac and Rho as in the extended model

|  | $\frac{\partial R}{\partial t} = A^{R}\left( \rho\right)\frac{R_{i}}{R_{tot}}-R+D\Delta R$ | Eqn. S1 |
| --- | --- | --- |
|  | $\frac{\partial\rho}{\partial t} = A^{\rho}\left( R \right)\frac{\rho_{i}}{\rho_{tot}}-\rho+D\Delta\rho$ | Eqn. S2 |
|  | $\frac{\partial R_{i}}{\partial t} = {- A}^{R}\left( \rho\right)\frac{R_{i}}{R_{tot}}+R+D_{i}\Delta R_{i}$ | Eqn. S3 |
|  | $\frac{\partial\rho_{i}}{\partial t} = {- A}^{\rho}\left( R \right)\frac{\rho_{i}}{\rho_{tot}}+\rho+D_{i}\Delta\rho_{i}$ | Eqn. S4 |

with

|  | $A^{R}\left( \rho\right)= {(b}_{R}+ \gamma_{R})(\frac{1}{1+\rho^{n}})$,  $A^{\rho}\left( R \right)={(b}_{\rho}+ \gamma_{\rho})(\frac{1}{1+R^{n}}).$ | Eqn. S5 |
| --- | --- | --- |

$A^{j}, b_{j}$ , $\gamma_{j}$ correspond to the activation rates of Rac, Rho, the initial activation rate and feedback components. n is the Hill coefficient. As a simplification, it is assumed that $b_{R}=b_{\rho}$ after initial triggering of the system by Rac activation. The parameter values are defined in S1 Table.

***2.2 Initial conditions***

The initial active and inactive Rac and Rho concentrations are as defined in S1 Table. In order to obtain comparable maximum concentrations for Rac after 10s as with the extended model, we used the initial polarization scheme for Rac below (which is different from the extended model’s initial polarization)

|  | $b_{R}=\left\{ \begin{aligned} 0.7+0.125x, &t\leq10s \\ 4, &t>10s \end{aligned} \right.$ | Eqn. S6 |
| --- | --- | --- |

The remaining parameter values can be found in Table 1 (see main text).

| **Parameter** | **Definition** | **Value** |
| --- | --- | --- |
| n | Hill coeff. of Cdc42-Rho mutual inhibition | 4 |
| γ_R_ | Rho-dependent Rac activation rate | 1 s^-1^ |
| γ_ρ_ | Rac-dependent Rho activation rate | 1 s^-1^ |
| D, D_i_ | diffusion coefficient of active, inactive Rac and Rho proteins | 0.01, 10 µm^2^ s^-1^ |

**S1 Table.** **Parameter values for the minimal model as used by Holmes et al.** [1]**.**


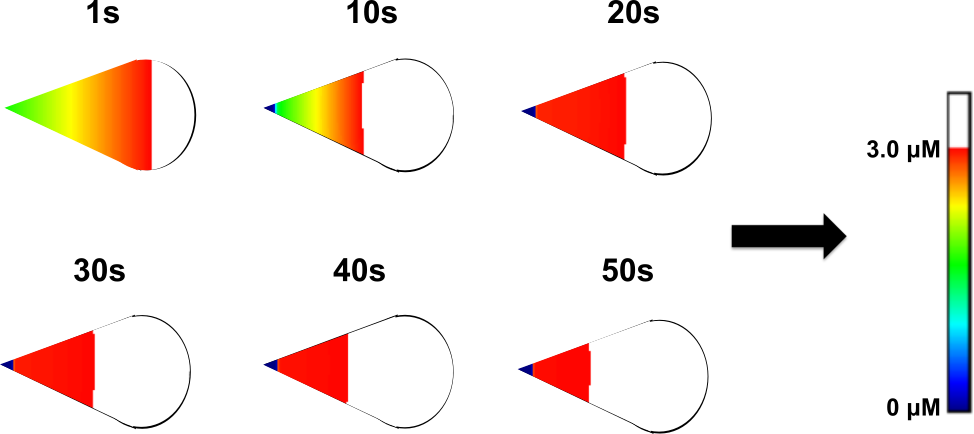


**S23 Fig.** **Spatiotemporal evolution of active Rac in a teardrop-shaped cell, for the minimal model. Regions where the active Rac concentration is above 3.0 µM are colored white.** The black arrow indicates the initial left-right direction of polarization


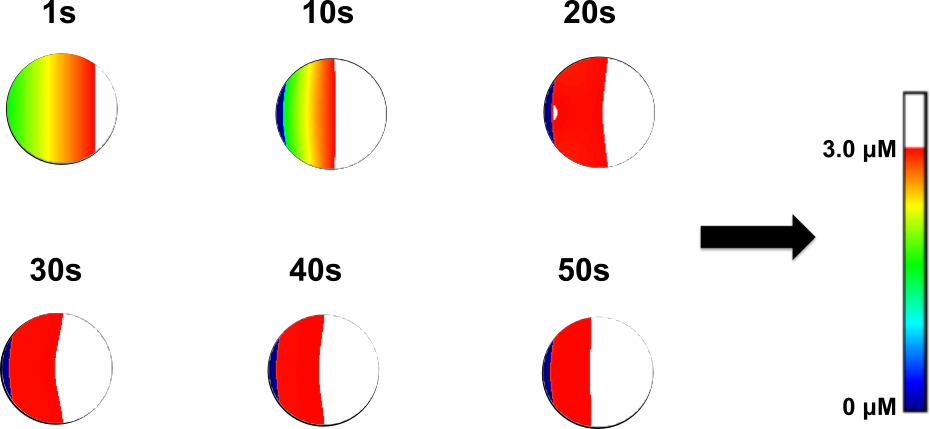


**S24 Fig.** **Spatiotemporal evolution of active Rac in a circular-shaped cell, for the minimal model. Regions where the active Rac concentration is above 3.0 µM are colored white.** The black arrow indicates the initial left-right direction of polarization


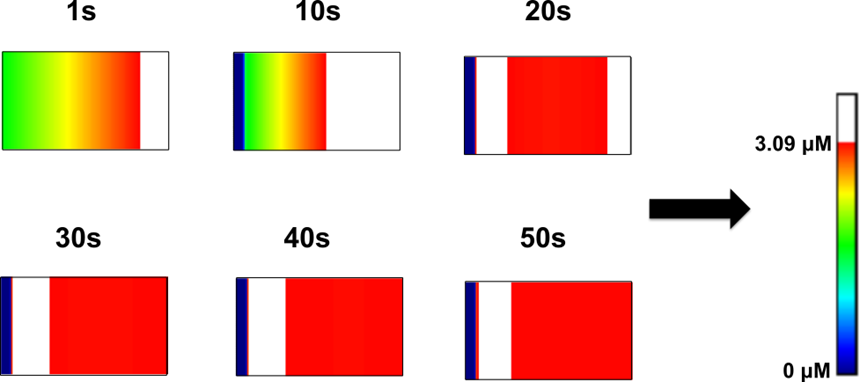


**S25 Fig.** **Spatiotemporal evolution of active Rac in a rectangular-shaped cell, for the minimal model.** Regions where the active Rac concentration is above 3.09 µM are colored white. The black arrow indicates the initial direction of polarization


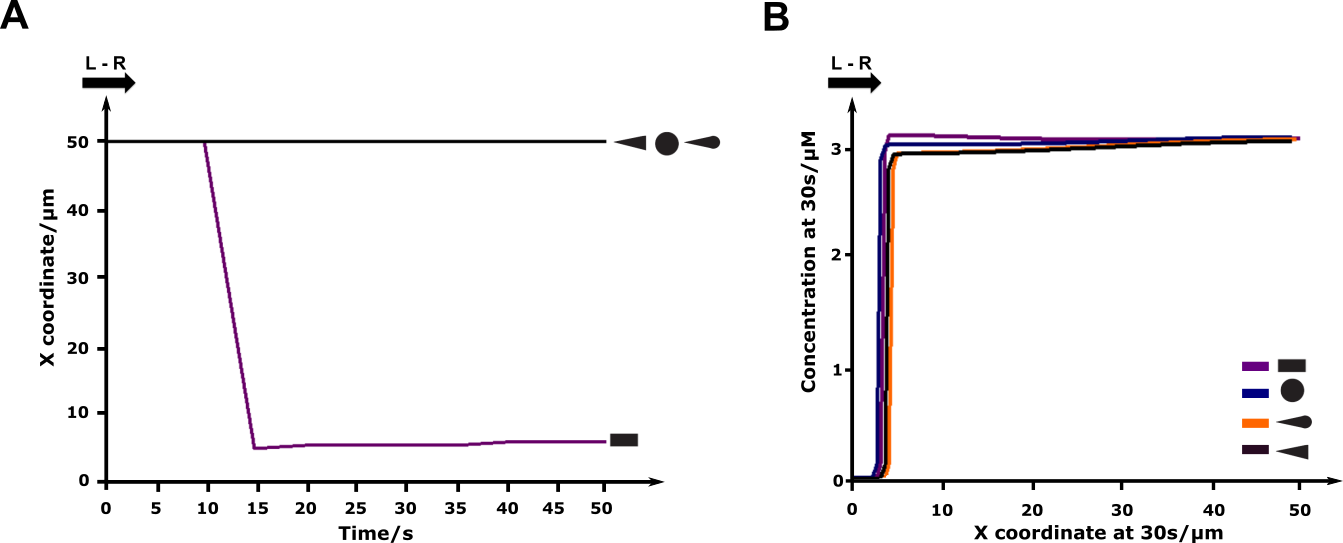


**S26 Fig.** **Cell shape influences polarization in the minimal model.** A) Temporal evolution of maximal active Rac for a cell stimulated in L-R direction. The triangle, teardrop and circle overlap. B) Spatial evolution of maximal Rac concentration at 30s, for a cell stimulated in the L-R direction


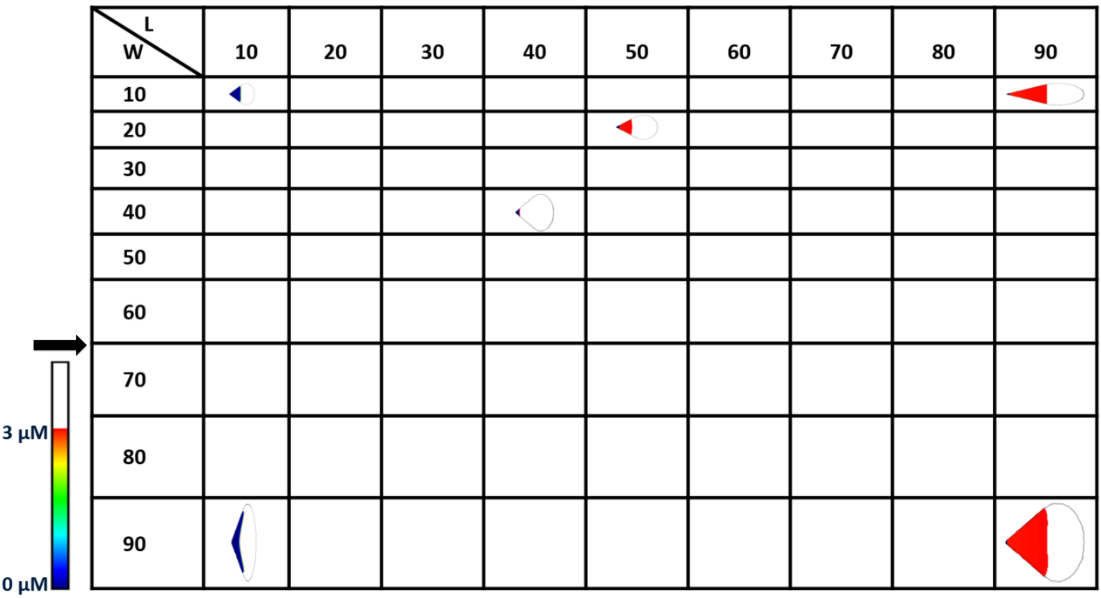


**S27 Fig.** **Cell size and aspect ratio do not alter polarization in the minimal model.** Polarization patterns of active Rac at different aspect ratios for the teardrop. L length, W width and simulation time of at 50s

**S28 Model code**

Teardrop extended model “Kerbai_PLoSCB_2020_teardrop_polarization_extended”: VCell mathmodel

MathDescription {

Constant _F_ 96485.3321;

Constant _F_nmol_ 9.64853321E-5;

Constant _K_GHK_ 1.0E-9;

Constant _N_pmol_ 6.02214179E11;

Constant _PI_ 3.141592653589793;

Constant _R_ 8314.46261815;

Constant _T_ 300.0;

Constant a1 1.25;

Constant a2 1.0;

Constant alpha 4.5;

Constant AreaPerUnitArea_plasma_membrane 1.0;

Constant Beta 0.3;

Constant cdc42_act_diffusionRate 0.1;

Constant cdc42_act_init_uM 1.0;

Constant cdc42_inact_diffusionRate 50.0;

Constant cdc42_inact_init_uM 1.4;

Constant cdc42_tot 2.4;

Constant d_cdc42 1.0;

Constant d_rac 1.0;

Constant d_rho 1.0;

Constant delta_P1 0.21;

Constant f 0.4;

Constant Ip 3.3;

Constant Ip1 10.5;

Constant Ir 0.5;

Constant k21 0.014;

Constant K_millivolts_per_volt 1000.0;

Constant k_PI3K 7.2E-4;

Constant k_PI5K 0.084;

Constant k_PTEN 0.432;

Constant KMOLE 0.001660538783162726;

Constant n 3.0;

Constant P1_diffusionRate 5.0;

Constant P1_init_uM 55.0;

Constant P2_diffusionRate 5.0;

Constant P2_init_uM 30.0;

Constant P3_diffusionRate 5.0;

Constant P3_init_uM 0.05;

Constant P3b 0.05;

Constant PI3K_init_uM 10.0;

Constant PI5K_init_uM 10.0;

Constant PI_init_uM 10.0;

Constant PTEN_init_uM 10.0;

Constant Rac_act_diffusionRate 0.1;

Constant Rac_act_init_uM 3.0;

Constant Rac_inact_diffusionRate 50.0;

Constant Rac_inact_init_uM 4.5;

Constant Rac_tot 7.5;

Constant Rb 3.0;

Constant Rho_act_diffusionRate 0.1;

Constant Rho_act_init_uM 1.25;

Constant Rho_b 1.25;

Constant Rho_inact_diffusionRate 50.0;

Constant Rho_inact_init_uM 1.85;

Constant Rho_tot 3.1;

Constant Voltage_plasma_membrane 0.0;

Constant VolumePerUnitVolume_cytosol 1.0;

Constant VolumePerUnitVolume_extracellular_space 1.0;

VolumeVariable Cell::cdc42_act

VolumeVariable Cell::cdc42_inact

VolumeVariable Cell::P1

VolumeVariable Cell::P2

VolumeVariable Cell::P3

VolumeVariable Cell::Rac_act

VolumeVariable Cell::Rac_inact

VolumeVariable Cell::Rho_act

VolumeVariable Cell::Rho_inact

VolumeRegionVariable Cell::PI

VolumeRegionVariable Cell::PI3K

VolumeRegionVariable Cell::PI5K

VolumeRegionVariable Cell::PTEN

Function Ic (((2.6 + (0.05 * x)) * (t <= 10.0)) + (2.95 * (t > 10.0)));

Function Cell::J_r0 ((Ic / (1.0 + ((Rho_act / a1) ^ n)) * (cdc42_inact / cdc42_tot) * ((1.0 - f) + (f * P3 / P3b))) - (d_cdc42 * cdc42_act));

Function Cell::J_r1 (((Ir + (alpha * cdc42_act)) * (Rac_inact / Rac_tot) * ((1.0 - f) + (f * P3 / P3b))) - (d_rac * Rac_act));

Function Cell::J_r2 (((Ip + (Beta * Rac_act)) / (1.0 + ((cdc42_act / a2) ^ n)) * (Rho_inact / Rho_tot)) - (d_rho * Rho_act));

Function Cell::J_r3 (( - k21 * P2) + (k_PI5K / 2.0 * (1.0 + (Rac_act / Rb)) * P1));

Function Cell::J_r4 ((k_PI3K / 2.0 * (1.0 + (Rac_act / Rb)) * P2) - (k_PTEN / 2.0 * (1.0 + (Rho_act / Rho_b)) * P3));

Function Cell::J_r5 (Ip1 - (delta_P1 * P1));

Function Cell::Size_cytosol (VolumePerUnitVolume_cytosol * vcRegionVolume('Cell'));

Function EC::Size_extracellular_space (VolumePerUnitVolume_extracellular_space * vcRegionVolume('EC'));

Function Cell_EC_membrane::Size_plasma_membrane (AreaPerUnitArea_plasma_membrane * vcRegionArea('Cell_EC_membrane'));

Function Cell_EC_membrane::sobj_Cell1_EC0_size vcRegionArea('Cell_EC_membrane');

Function Cell_EC_membrane::sobj_EC2_Cell1_size vcRegionArea('Cell_EC_membrane');

Function Cell::vobj_Cell1_size vcRegionVolume('Cell');

Function EC::vobj_EC0_size vcRegionVolume('EC');

Function EC::vobj_EC2_size vcRegionVolume('EC');

CompartmentSubDomain Cell {

BoundaryXm Flux

BoundaryXp Flux

BoundaryYm Flux

BoundaryYp Flux

PdeEquation cdc42_inact {

Rate - J_r0;

Diffusion cdc42_inact_diffusionRate;

Initial cdc42_inact_init_uM;

}

PdeEquation cdc42_act {

Rate J_r0;

Diffusion cdc42_act_diffusionRate;

Initial cdc42_act_init_uM;

}

PdeEquation Rac_inact {

Rate - J_r1;

Diffusion Rac_inact_diffusionRate;

Initial Rac_inact_init_uM;

}

PdeEquation Rac_act {

Rate J_r1;

Diffusion Rac_act_diffusionRate;

Initial Rac_act_init_uM;

}

PdeEquation Rho_inact {

Rate - J_r2;

Diffusion Rho_inact_diffusionRate;

Initial Rho_inact_init_uM;

}

PdeEquation Rho_act {

Rate J_r2;

Diffusion Rho_act_diffusionRate;

Initial Rho_act_init_uM;

}

PdeEquation P1 {

Rate ( - J_r3 + J_r5);

Diffusion P1_diffusionRate;

Initial P1_init_uM;

}

PdeEquation P2 {

Rate (J_r3 - J_r4);

Diffusion P2_diffusionRate;

Initial P2_init_uM;

}

PdeEquation P3 {

Rate J_r4;

Diffusion P3_diffusionRate;

Initial P3_init_uM;

}

VolumeRegionEquation PI5K {

UniformRate 0.0;

VolumeRate 0.0;

Initial PI5K_init_uM;

}

VolumeRegionEquation PI3K {

UniformRate 0.0;

VolumeRate 0.0;

Initial PI3K_init_uM;

}

VolumeRegionEquation PTEN {

UniformRate 0.0;

VolumeRate 0.0;

Initial PTEN_init_uM;

}

VolumeRegionEquation PI {

UniformRate 0.0;

VolumeRate - J_r5;

Initial PI_init_uM;

}

}

CompartmentSubDomain EC {

BoundaryXm Flux

BoundaryXp Flux

BoundaryYm Flux

BoundaryYp Flux

}

MembraneSubDomain Cell EC {

Name Cell_EC_membrane

BoundaryXm Value

BoundaryXp Value

BoundaryYm Value

BoundaryYp Value

JumpCondition cdc42_inact {

InFlux 0.0;

OutFlux 0.0;

}

JumpCondition cdc42_act {

InFlux 0.0;

OutFlux 0.0;

}

JumpCondition Rac_inact {

InFlux 0.0;

OutFlux 0.0;

}

JumpCondition Rac_act {

InFlux 0.0;

OutFlux 0.0;

}

JumpCondition Rho_inact {

InFlux 0.0;

OutFlux 0.0;

}

JumpCondition Rho_act {

InFlux 0.0;

OutFlux 0.0;

}

JumpCondition P1 {

InFlux 0.0;

OutFlux 0.0;

}

JumpCondition P2 {

InFlux 0.0;

OutFlux 0.0;

}

JumpCondition P3 {

InFlux 0.0;

OutFlux 0.0;

}

JumpCondition PI5K {

InFlux 0.0;

OutFlux 0.0;

}

JumpCondition PI3K {

InFlux 0.0;

OutFlux 0.0;

}

JumpCondition PTEN {

InFlux 0.0;

OutFlux 0.0;

}

JumpCondition PI {

InFlux 0.0;

OutFlux 0.0;

}

}

}

Teardrop minimal model “Kerbai_PLoSCB_2020_teardrop_polarization_minimal”: VCell mathmodel

MathDescription {

Constant _F_ 96485.3321;

Constant _F_nmol_ 9.64853321E-5;

Constant _K_GHK_ 1.0E-9;

Constant _N_pmol_ 6.02214179E11;

Constant _PI_ 3.141592653589793;

Constant _R_ 8314.46261815;

Constant _T_ 300.0;

Constant AreaPerUnitArea_plasma_membrane 1.0;

Constant b_Rho 4.0;

Constant gamma_Rho 1.0;

Constant I_Rac 1.0;

Constant I_Rho 1.0;

Constant K_millivolts_per_volt 1000.0;

Constant KMOLE 0.001660538783162726;

Constant n1 4.0;

Constant n2 4.0;

Constant Rac_act_diffusionRate 0.01;

Constant Rac_act_init_uM 3.0;

Constant Rac_inact_diffusionRate 10.0;

Constant Rac_inact_init_uM 4.5;

Constant Rac_tot 7.5;

Constant Rho_act_diffusionRate 0.01;

Constant Rho_act_init_uM 1.25;

Constant Rho_inact_diffusionRate 10.0;

Constant Rho_inact_init_uM 1.85;

Constant Rho_tot 3.1;

Constant Voltage_plasma_membrane 0.0;

Constant VolumePerUnitVolume_cytosol 1.0;

Constant VolumePerUnitVolume_extracellular_space 1.0;

VolumeVariable Cell::Rac_act

VolumeVariable Cell::Rac_inact

VolumeVariable Cell::Rho_act

VolumeVariable Cell::Rho_inact

Function Cell::b_Rac (((0.7 + (0.125 * x)) * (t <= 10.0)) + (4.0 * (t > 10.0)));

Function Cell::J_r1 ((((b_Rac + gamma_Rho) * (1.0 / (1.0 + (Rho_act ^ n1)))) * (Rac_inact / Rac_tot)) - (I_Rac * Rac_act));

Function Cell::J_r2 ((((b_Rho + gamma_Rho) * (1.0 / (1.0 + (Rac_act ^ n2)))) * (Rho_inact / Rho_tot)) - (I_Rho * Rho_act));

Function Cell::Size_cytosol (VolumePerUnitVolume_cytosol * vcRegionVolume('Cell'));

Function EC::Size_extracellular_space (VolumePerUnitVolume_extracellular_space * vcRegionVolume('EC'));

Function Cell_EC_membrane::Size_plasma_membrane (AreaPerUnitArea_plasma_membrane * vcRegionArea('Cell_EC_membrane'));

Function Cell_EC_membrane::sobj_Cell1_EC0_size vcRegionArea('Cell_EC_membrane');

Function Cell_EC_membrane::sobj_EC2_Cell1_size vcRegionArea('Cell_EC_membrane');

Function Cell::vobj_Cell1_size vcRegionVolume('Cell');

Function EC::vobj_EC0_size vcRegionVolume('EC');

Function EC::vobj_EC2_size vcRegionVolume('EC');

CompartmentSubDomain Cell {

BoundaryXm Flux

BoundaryXp Flux

BoundaryYm Flux

BoundaryYp Flux

PdeEquation Rac_inact {

Rate - J_r1;

Diffusion Rac_inact_diffusionRate;

Initial Rac_inact_init_uM;

}

PdeEquation Rac_act {

Rate J_r1;

Diffusion Rac_act_diffusionRate;

Initial Rac_act_init_uM;

}

PdeEquation Rho_inact {

Rate - J_r2;

Diffusion Rho_inact_diffusionRate;

Initial Rho_inact_init_uM;

}

PdeEquation Rho_act {

Rate J_r2;

Diffusion Rho_act_diffusionRate;

Initial Rho_act_init_uM;

}

}

CompartmentSubDomain EC {

BoundaryXm Flux

BoundaryXp Flux

BoundaryYm Flux

BoundaryYp Flux

}

MembraneSubDomain Cell EC {

Name Cell_EC_membrane

BoundaryXm Value

BoundaryXp Value

BoundaryYm Value

BoundaryYp Value

JumpCondition Rac_inact {

InFlux 0.0;

OutFlux 0.0;

}

JumpCondition Rac_act {

InFlux 0.0;

OutFlux 0.0;

}

JumpCondition Rho_inact {

InFlux 0.0;

OutFlux 0.0;

}

JumpCondition Rho_act {

InFlux 0.0;

OutFlux 0.0;

}

}

}

**Bibliography**

1. Holmes WR, Edelstein-Keshet L. Analysis of a minimal Rho-GTPase circuit regulating cell shape. Phys Biol. 2016;13: 046001. doi:10.1088/1478-3975/13/4/046001
